# A preliminary investigation on single nucleotide polymorphism rs2287622 of bile salt export pump gene in patients with chronic hepatitis C virus infection in Hunan, China

**DOI:** 10.1186/s12876-017-0594-9

**Published:** 2017-03-14

**Authors:** Jian-Hua Lei, Xu Yang, Xin-Qiang Xiao, Zi Chen, Feng Peng

**Affiliations:** Department of Infectious Diseases, the Second Xiangya Hospital, Central South University, No.139 Middle Renmin Road, Changsha, Hunan 410011 People’s Republic of China

**Keywords:** Single nucleotide polymorphism, Bile salt export pump, Hepatitis C virus, Chronic infection, China

## Abstract

**Background:**

European researchers have underscored associations between single nucleotide polymorphism (SNP) rs2287622 of the hepatobiliary bile salt export pump (BSEP) gene and the risk of hepatitis C virus (HCV) infection. The distributions of SNP rs2287622 are racially specific. This study was aimed to preliminarily investigate the distribution of BSEP gene SNP rs2287622 in the Han patients with chronic HCV-infection (CHC) in Hunan, China.

**Methods:**

BSEP gene SNP rs2287622 of 165 CHC patients, 99 patients with chronic hepatitis B virus infection (CHB) and 99 healthy individuals were analyzed by polymerase chain reaction-restriction fragment length polymorphism analysis and nucleotide sequencing.

**Results:**

The overall frequencies of the C allele of BESP gene SNP rs2287622 in the CHC patients, CHB patients and healthy individuals were 74.2, 72.7 and 74.2%, respectively (*P* > 0.05). The overall odds ratios (ORs) aiming at predicting CHC risk by comparing the ratios of the frequency distribution of alleles or genotypes in the CHC group with those in the non-CHC group had no statistical significance (*P* > 0.05). However, the CHC ORs of CC vs TT, TC vs TT and CC + CT vs TT among the individuals aged over 40 years were 2.680, 3.122 and 2.824 respectively (*P* < 0.05), and the higher risk did not relate to gender, HCV genotypes and presence of HCV-related liver cirrhosis.

**Conclusions:**

Among the Han individuals aged over 40 years in Hunan, China, genotype CC or CT of BSEP gene SNP rs2287622 may correlate with higher risk of CHC in comparison with genotype TT. Further study with a larger cohort is essential.

## Background

Hepatitis C is a global pandemic disease. About 185 million people are or have previously been infected with hepatitis C virus (HCV), most of whom develop chronic hepatitis. Besides, new infections are emerging [1]. Hepatitis C is more prevalent in East Asia, especially in China, with an estimated prevalence rate of 3.2% in the general population [2, 3]. Most HCV-infected persons are unaware that they are at risk for hepatitis C-related life-threatening diseases such as liver cirrhosis and hepatocellular carcinoma, whose incidences are predicted to rise in the coming decade [1, 2].

Although it remains unclear, host genetic diversity is believed to contribute to the susceptibility to HCV infection or HCV clearance in vivo. In recent years, researches on different related genetic variants have been published [4–11]. Since 2009, single nucleotide polymorphisms (SNPs) of the interleukin-28B gene have been identified as accurate predictors for therapy response and spontaneous clearance of HCV infection [4, 5]. Significant associations of estrogen receptor α or Toll-like receptor 7 genetic polymorphisms with HCV infection susceptibility or viral clearance among Han Chinese population have been reported [6, 7].

Since 2011, several European researches have underscored associations between a certain SNP, V444A (HGVS name: NM_003742.2: c.1331 T > C; ref SNP: rs2287622) in exon 13 of the hepatobiliary bile salt export pump (BSEP) gene, and the risk of HCV infection, progression of liver fibrosis and even the virological response during anti-HCV therapy [8–11]. But their findings need to be validated by investigation on extended population, especially on population of different races. So far, studies on the SNPs of BSEP gene in the chronic HCV-infected population have been rarely reported in China. The aim of the present study was to make a preliminary investigation on the distribution of SNP rs2287622 of BSEP gene in Han patients with chronic HCV-infection in Hunan, China.

## Methods

### Patients and control cohort

Random sampling was carried out on patients with confirmed diagnosis of chronic HCV infection in Liver Disease Research Center, the Second Xiangya Hospital, Central South University during 2012 and 2013. The sample size was estimated by the formula N = Z^2^ × P×(1-P)/E^2^. The annual amount of the HCV-infected patients visiting the center is about 1000. The frequency of allele C in exon 13 c.1331 of BESP gene in Chinese and Japanese is about 0.70 [12–14]. When the confidence level was set at 95% (Z_0.05_ = 1.96) and the sampling error at 10% (E = 0.10), the sample size worked out was 75 for each year. Additional 10% of the sample size was recruited, and finally 82 and 83 cases were respectively sampled from the patients in 2012 and 2013. All the 165 patients were of the Han nationality.

The diagnostic criteria for the chronic HCV infection (CHC) were based on the combination of clinical history, physical examination, imaging and laboratory data and/or histology. All of the patients finally enrolled in the study were positive for serum anti-HCV antibodies and had detectable serum HCV-RNA for at least 6 months. One hundred and seven of them had abnormal liver function indicated by elevated aspartate aminotransferase (AST) and alanine aminotransferase (ALT) before anti-HCV treatment. One hundred and thirty-three cases had ever received systematic anti-HCV therapy by combination of recombinant interferon-alpha (INF-α) and ribavirin (RBV). Twenty-six of them adopted common INF-α/RBV therapeutic scheme and 107 adopted polyethyleneglycolated INF-α (Peg-INF-α)/RBV therapeutic scheme. Twenty-six of the 165 CHC patients were diagnosed as HCV-related liver cirrhosis and 10 of them had been treated with adjusted low dose Peg-INF-α/RBV therapy.

A total of 99 healthy individuals of the Han nationality without evidences of hepatitis B virus (HBV) or HCV infections available at the Physical Examination Center of the hospital on two randomly chosen days (November 21th, 2012 and January 15th, 2013) served as the health control. Their blood specimens, clinical features and demographic data were collected.

On January 15th, 2013, 99 chronic hepatitis B (CHB) cases of Han nationality, including 30 CHB related liver cirrhosis cases, were randomly selected from the opening CHB case database in our center, severing as the CHB control. The database had collected the demographic data, clinical features and genomic DNA specimens of more than 1500 CHB patients visiting our center since 2010, all of whom had been HBV surface antigen (HBsAg) positive for over six months and had detectable serum HBV DNA when their genomic DNA samples were collected. Inclusion of hepatitis B patients into the controls was based on two facts. Firstly, HBV and HCV are both hepatotropic viruses and share similar transmission routes and clinical manifestations. Secondly, having CHB patients as controls is popular in professional researches on CHC, so is in the similar European research [10].

One hundred and sixty-five CHB and 99 CHB cases finally enrolled in the study were all confirmed to have no evidence of co-infection with hepatitis A virus, hepatitis E virus, Epstein-Barr virus, cytomegalovirus or human immunodeficiency virus, no detectable anti-delta antibodies, or no evidence of other chronic liver disease (autoimmune chronic liver disease, alcoholic liver disease, drug-induced liver injury, hemochromatosis, Wilson’s disease, or ɑ1-antitrypsin deficiency). None of the 165 CHC or 99 CHB cases was co-infected with HCV and HBV.

The collection of all the demographic data, clinical features and biological specimens including genomic DNA from the peripheral blood of the research objects fulfilled the requirements of medical ethics. The ethical review committees of the Second Xiangya Hospital of Central South University approved this study. Guidelines of the Declaration of Helsinki set by the committees were strictly followed. Written informed consent was obtained from all participants.

### Clinical features of the patients and control cohort

Serum ALT, AST and total bilirubin of the patients were measured by a 7600 Series automatic analyzer (Hitachi High-Tech Co., Japan) according to the manufacturer’s instructions. Serum anti-HCV was assessed by an enzyme linked immunosorbent assay (ELISA) diagnostic kit (Zhuhai Livzon Diagnostics Inc., China), and HBsAg was tested by another ELISA diagnostic kit (Shanghai Kehua Bio-engineering Co., Ltd., China) according to the manufacturer’s instructions. Quantitation of plasma HCV RNA was assessed in 7500 real-time PCR system (Applied Biosystems Inc., USA) by using HCV RNA quantitative fluorescence diagnostic kit (Sansure biotech Inc. Ltd, China), and the lower limit of the detection was 25 IU/mL. Quantitation of serum HBV DNA was assessed in StepOnePlus real-time PCR system (Applied Biosystems Inc., USA) by using HBV DNA quantitative fluorescence diagnostic kit (Sansure biotech Inc. Ltd, China), and the lower limit of the detection was 10 IU/mL.

Liver cirrhosis was diagnosed by liver biopsy, or FibroScan liver stiffness more than 13 kilopascal (kPa) together with Child-Turcotte-Pugh score more than 7, or FibroScan liver stiffness more than 13 kPa together with any two of the following criteria: the presence of ascites, hepatic encephalopathy, upper gastrointestinal bleeding, endoscopic detection of gastroesophageal varices, radiologic imaging of nodular liver or splenomegaly and peripheral blood platelet count below 100 × 10^9^/L in the absence of other explanations. Liver stiffness was measured by the FibroScan® 502 transient elastography device (Echosens, Paris, France), and the liver stiffness values were expressed in units of kPa, ranging from 2.5 to 75 kPa. Percutaneous liver histological examination was performed from the right lobe under real-time ultrasound guidance, and the inflammation grades and fibrosis stages of the biopsy samples were interpreted by two experienced hepatopathologists who were blinded to the clinical data.

Based on the assessment of hepatic encephalopathy grade, ascites, prothrombin activity, serum albumin and bilirubin levels, liver cirrhosis severity was assessed in the cirrhotic cases using a modified Child-Pugh's classification system [15, 16]: 5–6 points, grade A; 7–9 points, grade B; 10–15 points, grade C.

### Collection of genomic DNA from peripheral blood

About 5 mL peripheral blood was collected from each patient into elhylene diamine tetraacetic acid (EDTA)-containing vacationer tubes. Plasma was stored at −70 °C until HCV RNA loading analysis. Peripheral blood monouclear cells (PBMCs) were isolated from EDTA-treated blood by lymphocyte separation medium (Tianjin Haoyang Biological manufacture Co., Ltd., China) by centrifugation over density gradient. PBMCs were then washed three times with phosphate-buffered saline (pH =7.4), counted and stored at −70 °C for later detection.

Genomic DNA was isolated from a pellet of approximately 3–5 × 10^6^ PBMCs using EZHighTM-TG DNA Extraction Kit (Texas BioGene, Inc. USA) according to the manufacturer’s instructions. The concentrations of DNA samples were measured using the T6 spectrophotometer (Beijing Purkinje General Instrument Co., Ltd., China), then the DNA samples were diluted to 10 ng/μl.

### Confirmation of SNP rs2287622 genotypes by polymerase chain reaction-restriction fragment length polymorphism analysis (PCR-RFLP)

The sequences of the oligonucleotide primers using for PCR amplification of exon 13 of BSEP gene were 5′-CACACAGACACCGAGTATCAACACA-3′ (sense) and 5′-CAGGACAGTCTCAATGTATGCTACACCT-3′ (antisense).

PCR was performed in a total volume of 30 μl containing 60 ng genomic DNA, 0.3 pmol of each primer, 15 μl 2 × Taq MasterMix (0.05 U/μl Taq DNA polymerase, reaction buffer, 4 mM MgCl_2_, and 0.4 mM of each dNTP; Beijing ComWin Biotech Co., Ltd., China), 0.75 units Pfu DNA Polymerase (recombinant; Beijing ComWin Biotech Co., Ltd., China) and sterile deionized nuclease-free water. A PTC-100TM Programmable thermal controller (MJ Research, Inc., USA) was used for PCR with the following cycling conditions: initial denaturation at 94 °C for 3 min, followed by 35 cycles consisting of denaturation at 94 °C for 30 s, annealing at 60 °C for 30 s, extension at 72 °C for 30 s, and a final extension step at 72 °C for 5 min followed by cooling to 25° for 5 min. The PCR amplicon fragment was 333 base pairs in length.

10 μL PCR products were digested by HaeIII restriction endonuclease and buffer R (Fermentas Inc., USA) at 37 °C for 12 h, then the restriction digestion products for each were separated on 1.5% agarose gel stained with ethidium bromide for visualization on a ultraviolet transilluminatior. HaeIII digestion of allele CC yielded fragments of 127 and 206 base pairs, whereas PCR amplicons containing the allele TT polymorphism maintained 333 base pairs and amplicons containing the allele TC polymorphism yielded fragments of 127, 206 and 333 base pairs. All of the tests were performed in duplicate.

### Nucleotide sequencing

In order to confirm the accuracy of the detection results of SNP rs2287622 genotypes by PCR-FRLP, PCR was performed in triplicate on the genomic DNA samples of the 165 chronic HCV infection patients, 33 samples randomly chosen from the 99 chronic HBV infection patients and 33 from the 99 healthy individuals. The PCR products were purified with Wizard® SV Gel and PCR Clean-Up System (Promega Biotech Co., Ltd, USA) according to the manufacturer’s instructions. Direct sequencing of the purified products was performed by Shanghai Sangon Biotech Co., Ltd., China, with 3730xl DNA Analyzer and ABI BigDye® Terminator v3.1 Cycle Sequencing kit (Applied Biosystems Inc., USA). The sequences obtained were compared with the BSEP reference sequence derived from publicly available databases provided by NCBI (http://www.ncbi.nlm.nih.gov/) to confirm the SNP rs2287622 genotypes.

### Statistical analysis

All statistical analyses were performed with IBM® SPSS® Statistics version 20.0. The BSEP SNP rs2287622 genotypes and clinical features were compared between patients and control cohort. Chi-squared test, Analysis of Variance and Student *t*-test were used for comparisons between groups and Hardy-Weinberg equilibrium tests of the genotyping results. Allelic discrimination assay and association case–control analysis based on gender-, age-, HCV genotype- or liver cirrhosis-stratified analyses were performed to investigate the role of the BSEP SNP rs2287622 genotyping in the susceptibility to HCV infection, and odds ratios (ORs) were given with 95% confidence intervals (CIs). For all these tests, *P* value less than 0.05 was considered statistically significant.

## Results

### Demographic characteristics and clinical parameters of the study subjects

Overall, 165 patients with chronic HCV infection, 99 patients with chronic HBV infection and 99 healthy individuals were included in this study. A summary of demographic data and clinical parameters of the study subjects was given in Table 1. All of the participants were aged 18 years or above. Among the three groups, gender was equally distributed and average age and BMI was not significantly different. Serum ALT and AST levels were comparable between HCV-infected and HBV-infected patients.

**Table 1:** Demographic characteristics and clinical parameters of the study subjects

Parmeter	CHC	CHB	Healthy	*P* value
n	165	99	99	–
Gender male (n, %)	88 (53.3)	50 (50.5)	50 (50.5)	NS
Age, years (mean ± SD, range)	43.3 ± 11.9 (18–70)	43.5 ± 12.3 (22–67)	40.3 ± 12.2 (18–68)	NS
BMI (mean ± SD, range)	23.2 ± 3.9 (16.5–34.6)	22.6 ± 3.5 (18.4–33.9)	23.8 ± 3.4 (17.1–32.3)	NS
ALT, IU/L (median ± SD, IQR)	82.0 ± 62.0 (70.2)	94.1 ± 65.1 (83.6)	normal	NS^b^
AST, IU/L (median ± SD, IQR)	65.6 ± 40.4 (50.1)	77.4 ± 42.0 (68.0)	normal	NS^b^
HCV RNA level, log IU/mL (median ± SD, IQR)	6.08 ± 1.35 (1.51)	—	—	—
HBV DNA level, log IU/mL (median, IQR)	—	5.97 ± 1.21 (1.43)	—	—

*BMI* body mass index, *SD* standard deviation, *IQR* interquartile range, *NS* non-significant, *P* > 0.05

^a^The clinical data of the patients treated with anti-HCV or anti-HBV therapy were collected at the start point of the treatment, while data of the other patients were collected on the day when the genomic DNA were collected

^b^Comparisons of serum aminotransferase levels were carried out only between patients with chronic HCV infection and those with chronic HBV infection

Among the 26 cirrhotic CHC cases, 10 were assessed as Child-Pugh (C-P) grade A and were treated with low dose Peg-INF-α/RBV therapy. Nine cases with C-P grade B and seven cases with C-P grade C received symptomatic and supportive treatment other than Peg-IFN-α/RBV combination therapy. Among the 30 cirrhotic CHB cases, 12 were assessed as C-P grade A, 10 as grade B and 8 as grade C. The severity of liver cirrhosis was statistically comparable between cirrhotic CHC and cirrhotic CHB cases.

### Confirmation of SNP rs2287622 genotypes of BSEP gene by PCR-RFLP and nucleotide sequencing

**Fig. 1:**
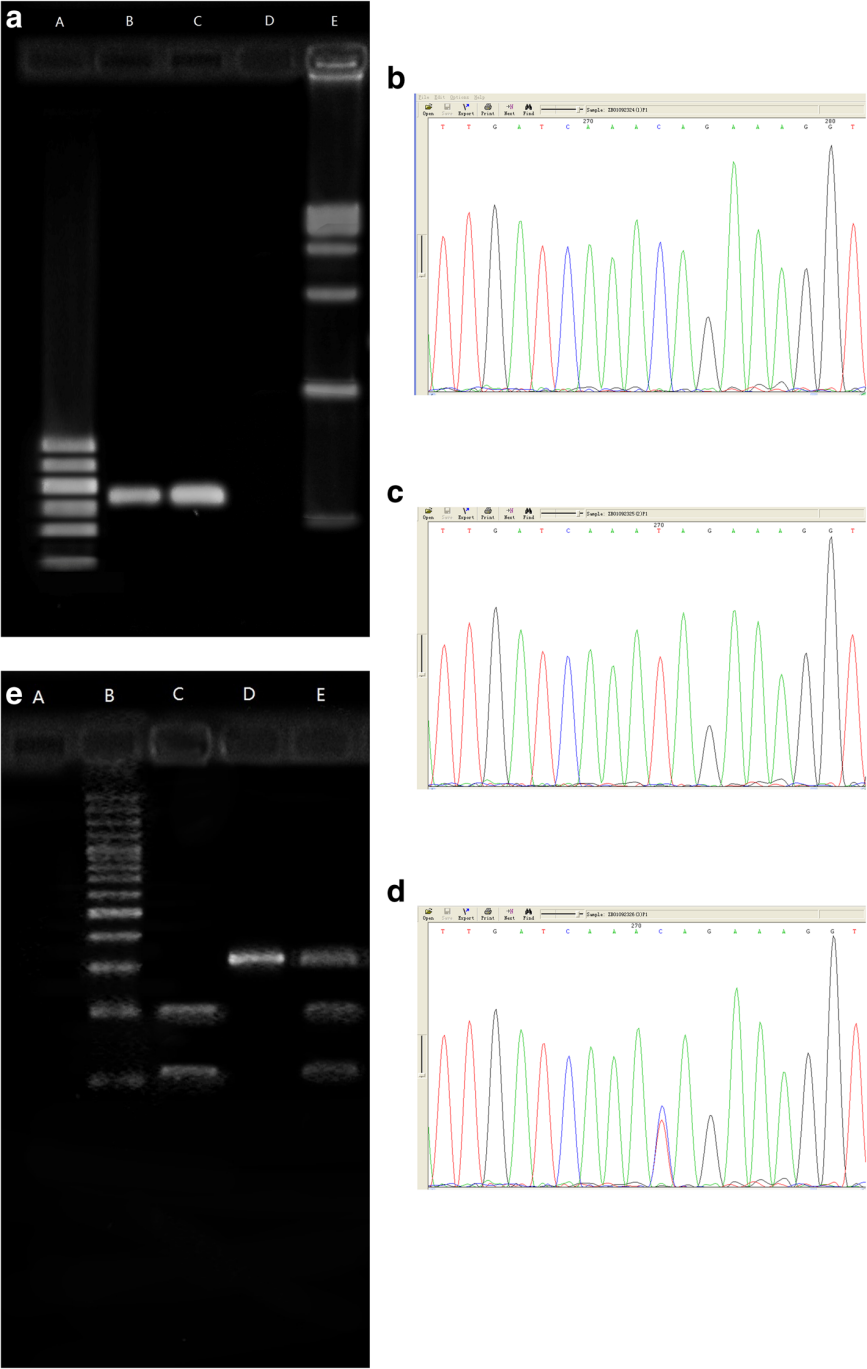
PCR amplification products of exon 13 of BSEP gene and confirmation of the SNP rs2287622 genotypes by nucleotide sequencing and RFLP. 1.1 amplification product of exon 13 of BSEP gene. **a**.DNA Marker I (Shanghai yuanye biotechnology Co., Ltd., China); **b**, **c**:cases; **d**:blank; **e**:DNA MarkerVI (Shanghai yuanye bio-technology Co., Ltd., China). 1.2 CC 1.3 TT 1.4 TC: Confirmation of rs2287622 genotypes by nucleotide sequencing. 1.5 Confirmation of rs2287622 genotypes by RFLP. **a**.blank; **b**.100bp DNA Ladder Marker (Beijing BLKW biotechnology Co., Ltd., China); **c**. rs2287622 genotype CC; **d**. rs2287622 genotype TT; **e**.rs2287622 genotype TC

The exon 13 and 5′ flanking region of BSEP gene of all samples were successfully amplified by PCR. RFLP analysis and nucleotide sequencing of the PCR products confirmed the SNP rs2287622 genotypes TT, CC and TC, as the codon of the 444th amino acid had three polymorphisms as GTC, GCC and heterozygote (see Fig. 1). Identification findings of the SNP rs2287622 genotypes by PCR-RFLP were completely consistent with the results by the triplicate PCR and nucleotide sequencing.

## Frequency distribution of BSEP rs2287622 alleles and genotypes

### Frequency distribution of BSEP rs2287622 genotypes

The frequency distribution of BSEP SNP rs2287622 in the research objects with different ages accorded with Hardy-Weinbery genetic equilibrium law (*P* > 0.05), suggesting that the cohorts were representative of the targeted population (Table 2).

**Table 2:** Frequency distribution of BSEP SNP rs2287622 genotypes in the research subjects of different age groups

Genotypes	18-years old			30-years old			40-years old			50-years old			60-years old		
	Non-CHC		CHC	Non-CHC		CHC	Non-CHC		CHC	Non-CHC		CHC	Non-CHC		CHC
	Health	CHB		Health	CHB		Health	CHB		Health	CHB		Health	CHB	
CC	16	10	12	19	14	18	11	14	37	9	13	20	5	6	7
TT	2	1	1	2	2	6	5	5	4	2	3	3	1	1	0
TC	6	5	10	9	9	11	6	7	17	4	6	11	2	3	8

### Frequency distribution of BSEP SNP rs2287622 alleles and genotypes on age stratification basis

The frequency distribution of BSEP SNP rs2287622 alleles and genotypes in the research subjects was described on age stratification basis (Table 2). The overall frequencies of the C allele of BESP gene SNP rs2287622 in the CHC patients, CHB patients and healthy individuals were 74.2, 72.7 and 74.2%, respectively, indicating no statistical difference (*P* > 0.05). The odds ratios (ORs) aiming at predicting CHC risk by comparing the ratios of the frequency distribution of allele C to T, of genotype CC, TC or CC + CT to TT, and of genotype TT, TC or TT + TC to CC in the CHC group with those in the non-CHC group had no statistical significance (*P* > 0.05) (Table 3). However, further data analysis found that when the research subjects were stratified into 18- and 40- years old age groups, the ORs of genotype CC to TT, TC to TT and CC + CT to TT in the 40- years old age group all had statistical significance (CC vs TT, OR = 2.680, 95%CI:1.037–6.924, *P* = 0.037;TC vs TT, OR = 3.122, 95%CI: 1.138–8.567, *P* = 0.024; CC+ CT vs TT, OR = 2.824, 95%CI:1.118–7.130, *P* = 0.023), indicating that homozygous and heterozygous presence of C allele was associated with CHC infection. Nevertheless, for the subjects aged 18- years, it was not the case.

**Table 3:** Frequency distribution of BSEP SNP rs2287622 alleles and genotypes in the research subjects on age stratification basis (n, %)

Alleles or genotypes		Overall			18- years old			40- years old		
		Non-CHC		CHC (*n* = 165)	Non-CHC		CHC (*n* = 58)	Non-CHC		CHC (*n* = 107)
		Health (*n* = 99)	CHB (*n* = 99)		Health (*n* = 54)	CHB (*n* = 41)		Health (*n* = 45)	CHB (*n* = 58)	
Alleles	C	147 (74.2)	144 (72.7)	245 (74.2)	85 (78.7)	62 (75.6)	81 (69.8)	62 (68.9)	82 (70.7)	164 (76.6)
	T	51 (25.8)	54 (27.3)	85 (25.8)	23 (21.3)	20 (24.4)	35 (30.2)	28 (31.1)	34 (29.3)	50 (23.4)
Genotypes	CC	60 (60.6)	57 (57.6)	94 (57.0)	35 (64.8)	24 (58.5)	30 (51.7)	25 (55.6)	33 (56.9)	64 (59.8)
	TT	12 (12.1)	12 (12.1)	14 (8.5)	4 (7.4)	3 (7.3)	7 (12.1)	8 (17.8)	9 (15.5)	7 (6.5)
	TC	27 (27.3)	30 (30.3)	57 (34.5)	15 (27.8)	14 (34.1)	21 (36.2)	12 (26.7)	16 (27.6)	36 (33.6)
Alleles analysis	Overall									
	Chi-square	0.172			2.391			2.517		
	*P* value	0.918			0.302			0.284		
	CHC vs Non-CHC									
	Chi-square	0.053			2.156			2.433		
	*P* value	0.817			0.142			0.119		
	Risk of CHC OR (95%CI) (C vs T)	1.040 (0.746–1.450)			0.677 (0.402–1.141)			1.412 (0.914–2.181)		
Genotypes analysis	Overall									
	Chi-square	2.379			2.340^a^			5.516		
	*P* value	0.666			0.674			0.238		
	CHC vs Non-CHC									
	Chi-square	2.157			1.892			5.388		
	*P* value	0.340			0.388			0.068		
	Risk of CHC									
	CC vs TT									
	Chi-square	0.779			1.395			4.355		
	*P* value	0.377			0.238			0.037		
	OR (95%CI)	1.377 (0.675–2.809)			0.508 (0.163–1.584)			2.680 (1.037–6.924)		
	CC + TC vs TT									
	Chi-square	1.270			0.957			5.146		
	*P* value	0.260			0.328			0.023		
	OR (95%CI)	1.488 (0.743–2.979)			0.580 (0.192–1.746)			2.824 (1.118–7.130)		
	TT + TC vs CC									
	Chi-square	0.166			1.595			0.264		
	*P* value	0.683			0.207			0.607		
	OR (95%CI)	1.091 (0.718–1.658)			1.530 (0.790–2.963)			0.866 (0.500–1.499)		
	TC vs TT									
	Chi-square	1.982			0.284			5.124		
	*P* value	0.159			0.594			0.024		
	OR (95%CI)	1.714 (0.806–3.645)			0.724 (0.221–2.377)			3.122 (1.138–8.567)		

*CHC* patients with chronic HCV infection, *CHB* patients with chronic HBV infection, *Healthy* Healthy individuals, *OR* odds ratio, *CI* confidence interval

^a^Continuity Corrected Chi-square

### Frequency distribution of BSEP SNP rs2287622 alleles and genotypes on gender stratification basis

Further analysis was conducted to explore the CHC risk of C allele in 40- years old subgroup. First, the gender differences of the frequency distributions of BSEP SNP rs2287622 alleles and genotypes were examined. The analysis showed that there were no statistical differences in the frequencies of the alleles and genotypes of BESP SNP rs2287622 between males and females, either for the overall population or for the 40- years old subgroup (all *P* >0.05) (Table 4).

**Table 4:** Frequency distribution of BSEP SNP rs2287622 alleles and genotypes on gender stratification basis

Alleles or genotypes		Overall							40- years old						
		Non-CHC				CHC		*P* value	Non-CHC				CHC		*P* value
		CHB		Healthy					CHB		Healthy				
		Male	Female	Male	Female	Male	Female		Male	Female	Male	Female	Male	Female	
Alleles	C	69	75	78	69	132	113	NS	41	41	20	42	78	86	NS
	T	31	23	24	27	44	41		19	15	16	12	22	28	
Genotypes	CC	26	31	33	27	51	43	NS	16	17	12	13	31	33	NS
	TT	7	5	6	6	7	7		5	4	5	3	3	4	
	CT	17	13	12	15	30	27		9	7	6	6	16	20	
*P* value			NS		NS		NS			NS		NS		NS	

*CHC* patients with chronic HCV infection, *CHB* patients with chronic HBV infection, *Healthy* Healthy individuals, *NS* non-significant

All *P* >0.05

### Frequency distribution of BSEP SNP rs2287622 alleles and genotypes on HCV genotype basis

As described in Table 5, the HCV genotypes distributed differently between CHC patients aged 18- years and those aged 40- years, and the proportion of type 1 HCV infection was statistically higher in CHC patients aged 40- years (Chi-square =14.035, *P* = 0.001). So the role of HCV genotypes in the higher presence of SNP rs2287622 allele C and genotypes CC and TC in CHC cases aged 40 years and above was explored. Further analysis was made to explore whether the higher presence of SNP rs2287622 allele C and genotypes CC and TC in CHC cases aged 40- years were associated with HCV type 1 infection. Therefore, the distribution of the frequencies of the genotypes of BESP gene SNP rs2287622 was compared among HCV type 1 infected patients, non HCV type 1 infected cases and those with unknown genotype. No significant difference was found, either for the overall, or for the 18- or 40- years age groups (all *P* >0.05) (Table 5).

**Table 5:** Frequency distribution of BSEP SNP rs2287622 genotypes on HCV genotype stratification basis (n, %)

HCV genotypes	Overall				18- years old				40- years old			
	n	CC	TT	CT	n	CC	TT	CT	n	CC	TT	CT
Type 1	59	32 (54.2)	6 (10.2)	21 (35.6)	14	6 (42.9)	1 (7.1)	7 (50.0)	45	26 (57.8)	5 (11.1)	14 (31.1)
Non type 1	20	13 (65.0)	2 (10.0)	5 (25.0)	14	9 (64.3)	2 (14.3)	3 (21.4)	6	4 (66.7)	0 (0.0)	2 (33.3)
Unknown	86	49 (57.0	6 (7.0)	31 (36.0)	30	15 (50.0)	4 (13.3)	11 (36.7)	56	34 (60.7)	2 (3.6)	20 (35.7)
Total	165	94 (57.0)	14 (8.5)	57 (34.5)	58	30 (51.7)	7 (12.1)	21 (36.2)	107	64 (59.8)	7 (6.5)	36 (33.6)
Chi-square	1.388^a^					2.617^a^				2.827^a^		
*P* value	0.846					0.624				0.572		

^a^Continuity Corrected Chi-square

### Frequency distribution of BSEP SNP rs2287622 alleles and genotypes on cirrhosis stratification basis in CHC and CHB patients

Among all the 165 CHC cases, 26 had clinical diagnosis of liver cirrhosis, among which 22 were older than 40 years. So the association of the alleles and genotypes of BESP SNP rs2287622 with liver cirrhosis was explored to find whether the predilection potential of C in CHC cases was due to its close relationship with liver cirrhosis. No significant differences in the frequencies of the alleles and genotypes of BESP gene SNP rs2287622 were found between CHC and CHB patients with and without liver cirrhosis (all *P* >0.05, Table 6).

**Table 6:** Frequency distribution of BSEP SNP rs2287622 alleles and genotypes on cirrhosis stratification basis in CHC and CHB patients (n, %)

Groups	Cirrhosis	Alleles		Genotypes			
		C	T	CC	TT	TC	Total
CHC	no	204	74	77 (55.4)	12 (8.6)	50 (36.0)	139 (100)
	yes	41	11	17 (65.4)	2 (7.7)	7 (26.9)	26 (100)
	Total	245	85	94 (57.0)	14 (8.5)	57 (34.5)	165 (100)
CHB	no	99	39	39 (56.5)	9 (13.0)	21 (30.4)	69 (100)
	yes	45	15	18 (60.0)	3 (10.0)	9 (30.0)	30 (100)
	Total	144	54	57 (57.6)	12 (12.1)	30 (30.3)	99 (100)

All *P* >0.05

## Discussion

BSEP is a hepatobiliary bile salt transporter on human hepatocellular canalicular membrane, one of the members of ATP-binding protein super family. Bile salts are transported to hepatocytes by Na^+ ^−taurocholate cotransporting polypeptide (NTCP) and sodium-independent organic anion transporting polypeptide (OATP), while they are transported from hepatocytes into bile canaliculus mainly by BSEP. BSEP plays an important role in the metabolism of hepatocellular bile acid [17, 18].

BSEP gene is located in the long arm of human chromosome 2, 2q24-31. It transcribes an mRNA of 5.5 kb. The expression of BSEP gene is mainly regulated by the farnesoid X receptor (FXR). Expression and activation of FXR in the cell is the key to the transcriptional activation of BSEP gene [18]. There are FXR binding elements in the promoter of BSEP gene. Many bile acid components, such as chenodeoxycholic acid (CDCA), lithocholic acid (LCA), deoxycholic acid (DCA) and cholic acid (CA), represent the native activators of FXR, among which CDCA can induce the strongest activation. The metabolic balance of bile acid in the hepatocytes is maintained by positive regulation on BSEP expression through activating FXR by bile acid.

In vitro experiments, studies have demonstrated that DCA and CDCA at physiological concentration increased the levels of HCV RNA and proteins up to five to ten folds, while the antagonist of the bile acid receptor or siRNA targeted at the gene of the bile acid receptor would reduce the bile acid-mediated increase of HCV RNA [19–21]. It has been reported that multiple bile acid components could increase HCV replication by activating FXR or EGFR/ERK pathway [21–23]. Meanwhile, they up-regulated apolipoprotein CII and activated a rate-limiting enzyme associated with the activation of the lipoprotein lipase. In these ways, they changed the course of HCV entering the host cells and the secretion of the virus particles from the host cells [21–23].

The clinical data indicated that elevated serum bile salt levels were significantly associated with increased risk of cirrhosis in patients with chronic HCV infection, and decreased sustained virological response (SVR) rate in patients treated with pegylated interferon-α and ribavirin. The mechanism may involve the block of the signal pathway of interferon, which is independent of FXR. Hydrophobic bile acids will inhibit Jak1- and Tyk2-phosphorylation, resulting in a decreased mRNA and protein expression of IFN-stimulated genes such as myxovirus resistance protein A (MxA) or dsRNA-activated protein kinase (PKR) thereby explaining interferon-ɑ resistance [24].

It has been reported that the polymorphism of BSEP gene might affect the expression and distribution of BSEP and transportation of bile acid, and eventually change the acid bile pool [17, 18, 25]. Studies on patients with intrahepatic cholestasis or idiopathic cholestasis during pregnancy indicated that polymorphism c.1331 T > C (p.V444A) (SNP rs2287622) in exon 13 of BSEP gene would lead to inhibition of the transportation of bile acid out of the hepatocytes [17, 25, 26].

In view of these, the effects of BSEP on the occurrence, progression and antiviral treatment response in chronic hepatitis C were studied. Europeans researches suggested that the polymorphism of BSEP V444A might be associated with the HCV infection development, liver fibrosis progression and long-term treatment response [8–11]. The few studies needed to be further supported by researches on extended population.

So far, no researches on the association between BSEP polymorphism and HCV infection in Chinese population were reported. Our preliminary study indicated that the frequency of allele C in BESP exon 13 c.1331 in Han patients with chronic HCV infection, chronic HBV infection and healthy individuals were 74.2, 72.7 and 74.2%, respectively, and the distributions of the alleles in the three groups were in accordance with Hardy-Weinberg equilibrium. The frequencies of allele C in the three groups were not statistically different. Our study suggested that the distributions of the polymorphism of BSEP SNP rs2287622 in the Han nationality in Hunan, China had different characteristics from those of Europeans. However, it had similar findings with the studies in the people without HCV infection in Chinese Taiwan and the mainland, and in a Japanese population, with frequencies of allele C in BESP exon 13 c.1331 of 75.6, 74.5 and 73.7%, respectively [12–14]. Similar genetic background in Chinese and Japanese supported the credibility of our findings.

Our research illustrated that the polymorphism of BSEP SNP rs2287622 was statistically correlated with chronic HCV infection in Han population aged 40 years and above. Interestingly, the correlation was not found in HCV patients aged 18–<40 years. Many gene polymorphisms or mutations, and their corresponding protein functions, and even the presence of clinical features, are age specific. The reasons for the age-related difference in frequency distribution of the alleles and genotypes of BSEP SNP rs2287622 were not included in this study. But further stratification analysis found that there were no statistical differences in the frequencies of the alleles and genotypes of BESP gene SNP rs2287622 between males and females, among CHC patients with different HCV genotypes, or between CHC patients with and without liver cirrhosis. It is speculated that BSEP might change the components of the bile acid pool in serum and liver cells to modify the chronicity process of HCV infection. Further study with a larger cohort of research subjects is essential.

## Conclusions

Among the Han individuals aged over 40 years in Hunan, China, genotype CC or CT of BSEP gene SNP rs2287622 may correlate with higher risk of CHC in comparison with genotype TT. Further study with a larger cohort is essential.

## Abbreviations

ALT: Alanine aminotransferase
AST: Aspartate aminotransferase
ATP: Adenosine triphosphate
BSEP: Bile salt export pump
CA: Cholic acid
CDCA: Chenodeoxycholic acid
CHB: Chronic HBV-infection
CHC: Chronic HCV-infection
CI: Confidence interval
DCA: Deoxycholic acid
DNA: Deoxyribonucleic acid
EDTA: Elhylene diamine tetraacetic acid
FXR: The farnesoid X receptor
HBsAg: HBV surface antigen
HBV: Hepatitis B virus
HCV: Hepatitis C virus
INF-α: Interferon-alpha
IQR: Interquartile range
LCA: Lithocholic acid
NS: Non-significant
OR: Odds ratio
PBMC: Peripheral blood monouclear cell
PCR: Polymerase chain reaction
RBV: Ribavirin
RFLP: Restriction fragment length polymorphism analysis
RNA: Ribonucleic acid
RT-PCR: Reverse transcription-PCR
SD: Standard deviation
SNP: Single nucleotide polymorphism
SVR: Sustained virological response
UTR: Untranslated region
